# Genetic Diversity and Environmental Adaptation Signatures of the Great Seahorse (*Hippocampus kelloggi*) in the Coastal Regions of the Indo-Pacific as Revealed by Whole-Genome Re-Sequencing

**DOI:** 10.3390/ijms26031387

**Published:** 2025-02-06

**Authors:** Wen-Xin Hao, Ying-Yi Zhang, Xin Wang, Meng Qu, Shi-Ming Wan, Qiang Lin

**Affiliations:** 1 College of Fisheries, Hubei Hongshan Laboratory/Key Lab of Freshwater Animal Breeding, Ministry of Agriculture and Rural Affairs/Engineering Research Center of Green Development for Conventional Aquatic Biological Industry in the Yangtze River Economic Belt, Ministry of Education, Huazhong Agricultural University, Wuhan 430070, China; haowenxin@webmail.hzau.edu.cn; 2 CAS Key Laboratory of Tropical Marine Bio-Resources and Ecology, South China Sea Institute of Oceanology, Chinese Academy of Sciences, Guangzhou 510301, China; zhangyingyi1996@gmail.com (Y.-Y.Z.); wangxin2014@scsio.ac.cn (X.W.); qumeng@scsio.ac.cn (M.Q.)

**Keywords:** *Hippocampus kelloggi*, whole-genome re-sequencing, genetic diversity, environmental adaptation, Indo-Pacific

## Abstract

The great seahorse (*Hippocampus kelloggi*) is one of the larger species within the seahorse group and is widely distributed in coastal areas of the Indo-Pacific. However, the natural resources of this species continue to decrease, rendering it a vulnerable species that faces a high risk of extinction. Therefore, there is an urgent need to conduct research on the genetic diversity of this species to protect its genetic resources. In this study, we conducted whole-genome re-sequencing (WGRS) on three *H. kelloggi* populations from the Red Sea (RS, n = 30), the Andaman Sea (AS, n = 13), and the South China Sea (SCS, n = 13), and a total of 1,398,936 high-quality single-nucleotide polymorphisms (SNPs) were identified. The results indicate that the average observed heterozygosity (*Ho*) and the average expected heterozygosity (*He*) for the RS, AS, and SCS populations are 0.2031 and 0.1987, 0.1914 and 0.1822, and 0.2083 and 0.2001, respectively. The three geographic populations exhibit a high degree of genetic differentiation with only a minimal gene flow between them. Consistently, in a population structure analysis, the three groups are also clearly distinguished, which is consistent with the results of the population differentiation coefficient. Demographic analyses revealed that the effective population size (*Ne*) of the SCS population underwent a dramatic bottleneck during the Last Glacial Maximum (LGM), followed by a substantial recovery, whereas the RS and AS populations maintained stable *Ne* values throughout this period. To investigate adaptive responses to climate change in the SCS population, we employed selective elimination analysis, which identified 21 candidate genes potentially involved in environmental adaptation. Of particular significance were *myo5a*, *hps4*, *znf385a*, *msh3*, and *pfkfb4*, which likely play crucial roles in the adaptive mechanisms of *H. kelloggi*. This comprehensive study not only illuminates the genetic diversity patterns of *H. kelloggi* but also provides a valuable foundation for future investigations into the species’ evolutionary adaptations.

## 1. Introduction

The great seahorse, *Hippocampus kelloggi*, is one of the larger species within the seahorse group, with a broad distribution across the Indo-Pacific region, encompassing the Red Sea off East Africa, the Indo-Australian Archipelago, and waters near Japan [1]. Unlike other seahorse species, *H. kelloggi* exhibits a unique trans-oceanic and trans-latitudinal distribution pattern, resulting in significant habitat variations among populations [2]. As a valuable ingredient in traditional Chinese medicine, *H. kelloggi* possesses considerable economic importance and diverse therapeutic properties, including anti-thrombotic and anti-aging effects [3]. However, due to habitat destruction and overfishing, wild populations of most seahorse species, including *H. kelloggi*, have experienced significant declines [4]. It is estimated that the population of *H. kelloggi* decreased by at least 30% between 2006 and 2016, resulting in its inclusion on the IUCN Red List of Endangered Species in 2017 [5]. While habitat destruction and overfishing remain major threats to *H. kelloggi* populations, climate change also poses a significant challenge to their survival. Decreased water temperature, ocean acidification, and changes in ocean currents can all negatively impact *H. kelloggi* habitats and their prey, thereby exacerbating population declines [6]. Understanding how *H. kelloggi* adapts to these climatic pressures is essential for devising effective conservation strategies. Studies have demonstrated that species’ environmental adaptability and evolutionary potential are intrinsically linked to their genetic diversity [7,8]. The assessment of genetic diversity within a species’ germplasm resources is fundamental for sustainable resource utilization and the maintenance of species–ecosystem coexistence [9]. Therefore, investigating the genetic diversity of *H. kelloggi* is crucial for protecting its genetic resources and developing effective conservation strategies.

Research on *H. kelloggi* remains limited due to its unique biological characteristics and conservation status. Existing studies primarily focus on morphological features [10,11] and habitat distribution [3,4,12], whereas population genetic studies are notably scarce. Seahorses are particularly intriguing subjects for studying marine organism dispersal patterns and geographical distribution [13,14] due to their restricted dispersal capabilities [15] and distinctive male brood pouch reproductive strategy [16], which limit genetic exchange between populations and create distinct population structures. Consequently, a whole-genome analysis of *H. kelloggi* promises to provide comprehensive insights into the genetic relationships and biogeographical evolution patterns of seahorse populations across the Indo-Pacific region.

To date, previous population genetics studies on seahorses have predominantly relied on sparse molecular markers or concentrated on a limited number of conserved genetic fragments [17,18,19]. With the rapid advancement of sequencing technologies, researchers can now efficiently and precisely identify genetic variations across the entire genome. The emergence of high-throughput sequencing technologies has particularly revolutionized genomic and molecular biological research, enabling unprecedented insights into genetic architecture [20]. Currently, whole-genome re-sequencing (WGRS) and single-nucleotide polymorphism (SNP) loci have been widely used in the genetic research of aquatic species. WGRS can detect high-density SNPs and Indels across the entire genome, thereby exploring the genetic background, environmental adaptation, and evolutionary process of target populations or identifying candidate genes associated with important traits [21]. For instance, Zhao et al. [22] employed WGRS to investigate the environmental adaptability of *Sillago sinica* across diverse climatic and geographical regions. Similarly, Li et al. [2] conducted a comprehensive study combining a de novo genome assembly of *Hippocampus erectus* with 358 re-sequenced genomes from 21 species to examine global seahorse populations. Their research revealed the species’ center of origin, dispersal patterns, and spatiotemporal migration pathways, while also illuminating the evolutionary mechanisms underlying trait diversification during population differentiation, thereby establishing a crucial theoretical framework for seahorse population genetics.

In this study, we aim to gain insight into the genetic background of *H. kelloggi* by performing high-depth whole-genome re-sequencing on 56 individuals from three different regions in the Indo-Pacific area. While previous research on *H. kelloggi* has primarily relied on traditional molecular markers, whole-genome-level population genetic analyses of this species remain scarce. Our research specifically focused on examining genetic differentiation patterns, population structures, and adaptive evolutionary characteristics among these three populations. The results of this study will promote resource management and provide valuable genetic markers and a theoretical basis for the conservation and improvement of the germplasm of *H. kelloggi*.

## 2. Results

### 2.1. Variant Discovery and SNPs Annotation

**Figure 1:**
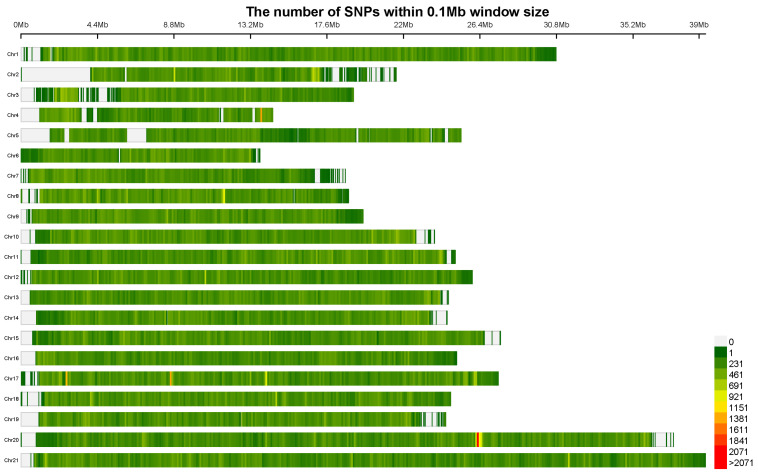
The genetic information of *Hippocampus kelloggi*. The identification of high-quality SNPs and their distribution across 21 chromosomes of *H. kelloggi*. The gradient colors from green to red indicate an increase in SNP density within 0.1 Mb interval.

In each sequencing library of three populations in the Red Sea (RS), the Andaman Sea (AS), and the South China Sea (SCS), an average of 84.89 million, 84.7 million, and 97.6 million high-quality clean reads were obtained. The average mapping rate of successful alignments to the reference genome of *H. kelloggi* was >97%, and the average sequencing depth reached 23× (Table S1). A total of 66,939,708 SNPs were obtained from all the analyzed samples. After annotation, 40.04% (26,657,611) of these SNPs were located in intergenic regions, 46.987% (31,282,950) were located in intronic regions, and only 3.918% (2,608,787) were located in exonic regions. Among all exonic variants, 54.95% (1,433,592) were synonymous mutations and 44.29% (1,155,340) were nonsynonymous mutations (Table 1). After stringent quality control, 1,398,936 high-quality SNPs were retained for a subsequent population genetic diversity analysis (Figure 1). These SNPs are relatively evenly distributed across the 21 chromosomes, although slight deletions are observed at the ends of some chromosomes.

### 2.2. Population Genetic Diversity Analysis

To further understand the genetic diversity of the natural populations of *H. kelloggi* in three regions, we calculated several metrics, including the inbreeding coefficient (*Fis*), average observed heterozygosity (*Ho*), average expected heterozygosity (*He*), polymorphism information content (*Pic*), nucleotide diversity ratios (*π*-*ratio*), effective number of alleles (*Ae*), and observed number of alleles (*Ao*) (Table 2). Our analysis shows that the *Fis* values for the three populations range from −0.0251 to −0.0082, all of which are negative, indicating the absence of inbreeding in these populations. Among the three populations, the *Ho* ranges from 0.1914 (AS) to 0.2083 (SCS), while the *He* ranges from 0.1822 (AS) to 0.2001 (SCS). In all populations, the *Ho* is higher than the *He*. The *Pic* ranges from 0.1534 (AS) to 0.1673 (RS), and the *π*-*ratio* ranges from 0.3269 (RS) to 0.3677 (SCS), indicating a moderate level of genetic diversity. Compared to the genetic diversity indices of the three *H. kelloggi* populations, it was found that, in all three populations, the *Ho*, *He*, and *Pic* are lowest in the AS, indicating lower genetic diversity, while the SCS has the highest values for the *Ho*, *He*, *Ae*, and *π*-*ratio* among the three populations, indicating higher genetic diversity.

### 2.3. Genetic Divergence and Population Structure Analysis of Three Populations

Based on whole-genome SNPs, the population differentiation of the RS, AS, and SCS populations was studied. The genetic differentiation between the three populations is relatively high, with a moderate differentiation between the RS and AS populations (pairwise fixation index (*Fst*) = 0.2371) and a high differentiation between the SCS and both the RS and AS populations (*Fst* = 0.3222 and 0.3183) (Table 3).

The genetic flow between the three populations was measured based on the number of effective migrants (*Nm*) value. The results show that there is low gene flow between these three populations, with a relatively limited genetic exchange (*Nm* < 1) (Table 3). The gene flow demonstrates a population differentiation similar to *Fst*, with the SCS population exhibiting slightly higher genetic differentiation compared to the RS and AS populations, as indicated by the smaller *Nm* value.

**Figure 2:**
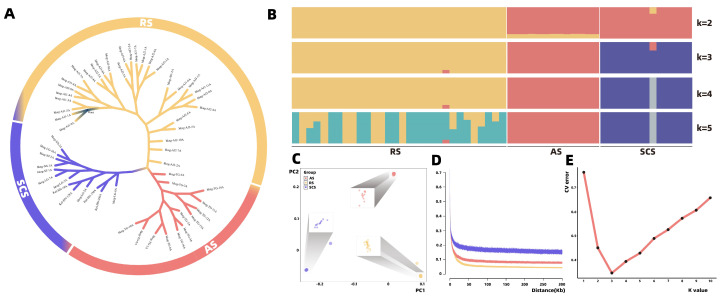
Population structure analyses of *H. kelloggi*. (**A**) A phylogenetic tree of the three analyzed populations based on genotype data. The blue, red and yellow backgrounds represent individuals in the SCS, AS, RS population, respectively. (**B**) A population structure map for K = 2~5. (**C**) Population structure revealed by PCA. The blue, red and yellow dots represent individuals in the SCS, AS and RS populations, respectively. (**D**) The decay of linkage disequilibrium in the three experimental populations. The *X*-axis represents physical location. The *Y*-axis represents the *LD* value (*r*^2^). The blue, red and yellow line represent the SCS, AS and RS populations, respectively. (**E**) The error rate of the cross validation (CV) for K = 1~10 (K value represents the number of subgroups of the population).

The Principal component analysis (PCA) results show that all analyzed samples are significantly clustered into three distinct clusters on the coordinate axis. Notably, for the SCS population, there is an outlier sample (Figure 2C). The phylogenetic tree analysis also confirms the distribution of similar samples, with the samples from the three populations being distinctly separated in the phylogenetic tree (Figure 2A).

In the population structure analysis, the test values for cross-validation error (CV error) were set from 1 to 10. When the CV error reached the minimum value, the optimal K value was observed (K = 3). This indicates that the 56 analyzed samples are most likely to be divided into three subgroups which clearly separate the populations from the three sampling sites (Figure 2B,E).

### 2.4. Correlation Analysis of Protein and mRNA

Using the *r*^2^ of adjacent pairs of SNPs to represent the linkage disequilibrium (*LD*) decay of the three populations. The maximum values of *r*^2^ in the SCS, AS and RS populations are similar, with the maximum average *r*^2^ values after smoothing being calculated as 0.6701, 0.6811 and 0.6633, respectively. The *r*^2^ for the AS and RS populations weakened to 0.0809 and 0.0452, respectively, at 300 kb. Within the short distance of 0–100 kb, the RS population shows the fastest rate of *r*^2^ decay, followed by the AS and SCS populations (Figure 2D).

### 2.5. Trends in Historical Effective Population Size

**Figure 3:**
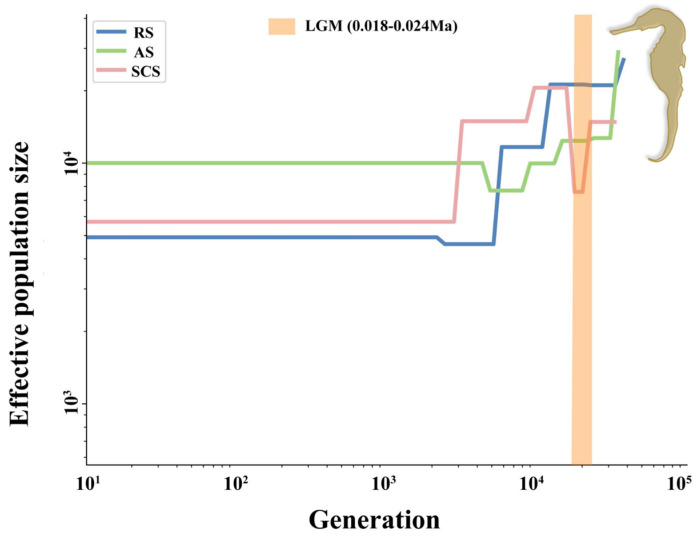
Demographic history of the three *H. kelloggi* populations in this study. Pink, green and blue dots represent individuals in the SCS, AS, RS populationd, respectively. The orange background represents the period of the Last Glacial Maximum (LGM).

In the historical effective population size variation graphs derived from the SMC++ method, the horizontal lines depict the temporal trends of *Ne*. Changes in *Ne* reflect alterations in the number of ancestral lineages within the population, thereby indicating population contraction, expansion, or stability [23]. Overall, the results of the SMC++ analysis revealed that the effective populations of *H. kelloggi* in the three regions have undergone a continuous decline for millions of years. Specifically, the *Ne* of the three populations began to diverge around 0.03 Mya and then stabilized around 2 Kya (Figure 3). It is worth noting that the *Ne* of the SCS population experienced a significant bottleneck effect during the Last Glacial Maximum (LGM, 0.18–0.24 Mya), with a rapid decline in population size, followed by a brief period of stability and then rapid expansion. In contrast, the *Ne* of the AS and RS populations remained stable during the LGM. This may imply the process of adaptation of the SCS population from intolerance to extreme cold environments to adaptation.

### 2.6. Candidate Genes Under Selection

**Figure 4:**
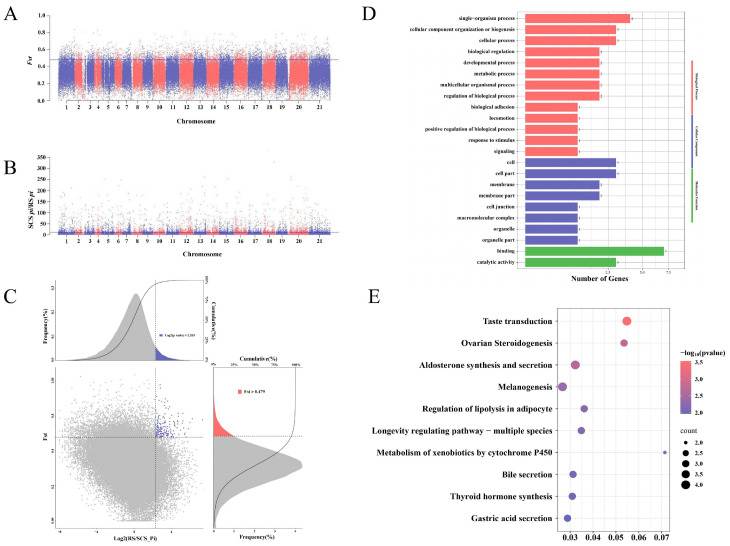
Candidate gene exploration and enrichment analysis of *H. kelloggi* from the SCS population (SCS vs. RS). (**A**) A plot of the moving average *F_ST_* (SCS vs. RS) values of the SNPs per chromosome. The blue line indicates the significant threshold for identifying putative selection regions (top 5 *F_ST_* = 0.479, *p*-value < 0.05). (**B**) Distribution of the *π*-*ratio* (SCS/RS) on 21 chromosomes. The blue line indicates the significant threshold for identifying putative selection regions (top 5 log2(*π*-*ratio* SCS/RS) = 2.265, *p*-value < 0.05). (**C**) The distribution of the log2 (*π*-*ratio*) and *F_ST_*. The RS population is the control group and the SCS population is the selection group. (**D**,**E**) Results of the GO and KEGG enrichment analysis of selected genes in the SCS population.

**Figure 5:**
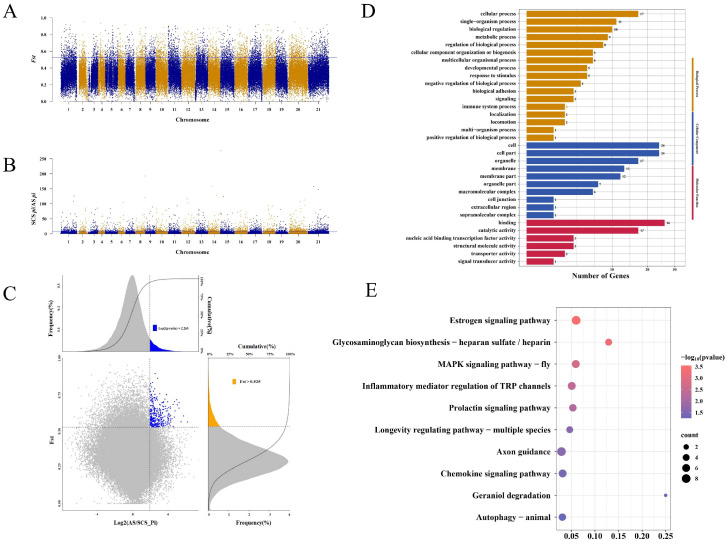
Candidate gene exploration and enrichment analysis of *H. kelloggi* from the SCS population (SCS vs. AS). (**A**) A plot of the moving average *F_ST_* (SCS vs. AS) values of SNPs per chromosome. The blue line indicates the significant threshold for identifying putative selection regions (top 5 *F_ST_* = 0.525, *p*-value < 0.05). (**B**) Distribution of the *π*-*ratio* (SCS/AS) on 21 chromosomes. The blue line indicates the significant threshold for identifying putative selection regions (top 5 log2(*π*-*ratio* SCS/AS) = 1.937, *p*-value < 0.05). (**C**) Distribution of the log2 (*π*-*ratio*) and *F_ST_*. The AS population is the control group and the SCS population is the selection group. (**D**,**E**) Results of the GO and KEGG enrichment analysis of selected genes in the SCS population.

In order to study the genetic adaptation selection of *H. kelloggi* in different geographical regions, we employed a comprehensive method combining the *Fst* and *π*-*ratio* to identify genomic selection regions closely related to environmental adaptivity (Figure 4A–C and Figure 5A–C). Considering the distinct historical effective population dynamics between the SCS population and the AS and RS populations, we used the AS and RS populations as reference groups and identified selected genes in the selected SCS population to explore the genetic traces of the LGM historical event.

Compared to the RS population (πRS/πSCS), only 99 genes displaying the selected features were identified in the SCS population. A Gene Ontology (GO) enrichment analysis showed that these genes were significantly enriched in 105 GO terms (Figure 4D). The major enriched GO terms included the single-organism process (GO: 0044699) and “cellular process” (GO: 0009987) in biological process, and the “membrane part” (GO: 0044425) and “membrane” (GO: 0016020) in cellular component, “binding” (GO: 0005488) and “catalytic activity” (GO: 0003824) in molecular function. A Kyoto Encyclopedia of Genes and Genomes (KEGG) enrichment analysis yielded a total of 31 significant biological pathways (Figure 4E); the top five biological pathways were “taste transduction” (ko04742), “ovarian steroidogenesis” (ko04913), “aldosterone synthesis and secretion” (ko04925), “melanogenesis” (ko04916), and “regulation of lipolysis in adipocyte” (ko04923).

Compared to the AS population (πAS/πSCS), 278 genes were identified as being under selection pressure in the SCS population. A GO enrichment analysis indicated that these genes were significantly enriched in 110 GO terms (Figure 5D). The main enriched GO terms include “cellular process” (GO:0009987) and “single-organism process” (GO:0044699) in biological process, “cell” (GO:0005623) and “cell part” (GO:0043226) in cellular component, and “binding” (GO:0005488) and “catalytic activity” (GO:0003824) in molecular function. In the KEGG enrichment analysis, a total of 10 biological pathways were significantly enriched (Figure 5E), with the top five biological pathways being “Estrogen signaling pathway” (ko04915), “Glycosaminoglycan biosynthesis—heparan sulfate/heparin” (ko00534), “MAPK signaling pathway—fly” (ko04013), “Inflammatory mediator regulation of TRP channels” (ko04750), and “Prolactin signaling pathway” (ko04917).

We identified 21 overlapping genes between the selected regions of the SCS vs. AS and the SCS vs. RS. These genes were viewed as potential genes associated with environmental adaptation for the SCS population (Table 4). This includes some genes that may function in animals’ adaptation to climate change, including myosin VA (*myo5a*) and HPS4 biogenesis of lysosomal organelles complex 3 subunit 2 (*hps4*) related to melanin synthesis; zinc finger protein 385A (*znf385a*) and mutS homolog 3 (*msh3*) related to genetic information repair and apoptosis; and 6-phosphofructo-2-kinase/fructose-2,6-biphosphatase 4 (*pfkfb4*) related to hypoxia adaptation. These genes may play a significant role in the process of climate adaptation in the SCS population.

## 3. Discussion

### 3.1. Genetic Diversity of H. kelloggi

Genetic diversity is an inherent manifestation of a species’ evolutionary potential and adaptive capacity. As a vulnerable species facing a high risk of extinction, evaluating the genetic diversity of *H. kelloggi* and comparing it with its closely related species holds significant importance for understanding its evolutionary history and informing conservation strategies. Among the important indicators for assessing genetic diversity are *Ho*, *He*, and *π*-*ratio*. Our study calculated these parameters for three natural populations of *H. kelloggi* from different sea areas using WGRS data. The results revealed *Ho* values ranging from 0.1914 to 0.2083, *He* values ranging from 0.1822 to 0.2001 and *π*-*ratio* values ranging from 0.3269 to 0.3677. Notably, the *Ho* values exceeded *He* values in all three populations, indicating an excess of heterozygotes. When compared with other fish populations analyzed using WGRS data, these values represent low to moderate levels of genetic diversity [24,25,26]. Previous studies on seahorse species have reported varying levels of genetic diversity. For instance, Lazic et al. [27] analyzed the genetic diversity of the long-snouted seahorse (*Hippocampus guttulatus*) in the Mediterranean region using eight microsatellite loci, reporting *Ho* values ranging from 0.23 to 0.44 and *He* values from 0.30 to 0.54. The comparatively lower values observed in our study populations might be attributed to either methodological differences in genetic variant detection or could indicate genuinely lower genetic variation in *H. kelloggi* populations.

The genetic diversity of the SCS population are all at the highest level among the three populations. However, in the linkage disequilibrium (*LD*) analysis, the decay rate of *LD* in the SCS population is the slowest among the three populations and the *LD* coefficient is the highest. The historical analysis of the population dynamics seems to support the results of the *LD* analysis. After experiencing a bottleneck effect during the LGM, the SCS population rapidly expanded, indicating that this was possibly due to the positive selection of the SCS population to adapt to the cold climate. Research has shown that, if a population is under positive selection, due to the effects of genetic linkage, the frequency of the surrounding loci linked to advantageous loci will rapidly increase, leading to a higher linkage between genetic variations [28]. However, in the context of high *LD* coefficients, the high genetic diversity of the SCS population may be due to various reasons. Firstly, the sampling points within the SCS population are relatively dispersed, which may result in a more complex genetic background within the population. Additionally, we suppose that there may have been an introgression of genetic information from new populations during the rapid expansion period of the SCS population. However, a strong *LD* linkage has already been established by the indigenous population, and even if genetic diversity has been restored within a short time, the previously high *LD* linkage may still be retained for a period.

### 3.2. Population Structure of H. kelloggi

This study investigated the genetic structure of three *H. kelloggi* populations through structure, phylogenetic, and principal component analyses. While marine ecosystems generally exhibit lower dispersal barriers compared to terrestrial ecosystems [29,30], and many marine fish species demonstrate long-distance dispersal capabilities [31,32], seahorses present a unique case. Their weak swimming abilities and reliance on drifting while entwined with floating objects result in localized living patterns [33]. This limited dispersal capacity, combined with geographical isolation, has led to a reduced gene flow and high genetic differentiation among *H. kelloggi* populations, as evidenced by our findings.

Our analyses provide multiple lines of evidence supporting this population structure. The principal component analysis, phylogenetic analysis, and population structure analysis all demonstrate clear differentiation among the three geographical populations, indicating distinct population stratification. Notably, while sampling points within the SCS population are relatively scattered along the coastline, they show no prominent genetic differentiation (Figure S1), suggesting that *H. kelloggi* can achieve medium to long-distance dispersal along nearshore ocean currents.

A comparative analysis of the genetic structures of the three populations reveals the influence of Chinese coastal ocean currents on *H. kelloggi* dispersal patterns. Seasonal oceanic circulation maintains genetic connectivity between geographically dispersed communities along the Chinese coastal area [9,34]. The Kuroshio Current and its tributaries create a barrier between the open ocean and coastal waters [9], while the Indo-Pacific Archipelago serves as a geographical barrier affecting oceanic circulation between the Indian and Pacific Oceans [35,36]. This has resulted in a relatively independent and isolated environment along the coast of China. Consequently, although the SCS population is geographically closer to the AS population, it exhibits a relatively high *Fst* value compared to both the AS and RS populations. In contrast, the genetic differentiation between the AS and RS populations is lower.

These findings suggest that the three natural populations of *H. kelloggi* have evolved into relatively independent genetic lineages within their respective habitats [27]. Such genetic structural characteristics indicate that long-term geographical isolation may have led to differential adaptation among these populations.

### 3.3. Historical Demography and Environmental Adaptation

According to our observations, the *Ne* of *H. kelloggi* has shown a significant decreasing trend across all three geographical regions over the past million years. Similar dynamic changes have been documented in other coastal species, including *Sillago sinica* [22] and *Mytilus coruscus* [9]. This decline in *Ne* can primarily be attributed to historically extreme environmental events and climate change-related stressors. The oscillation between glacial and interglacial periods has led to continuous fluctuations in ocean temperatures, imposing greater survival pressure on coastal species [37]. Additionally, human activities have emerged as a contributing factor to coastal biological population decline [38]. Notably, our analysis revealed distinct patterns in *Ne* dynamics across populations. The SCS population experienced a significant decline during the LGM, followed by a rapid rebound after a brief stabilization period, ultimately exceeding its original *Ne* level. In contrast, the AS and RS populations maintained stable *Ne* values during the LGM. This pattern suggests an adaptive process in the SCS population in response to severe environmental stress, potentially indicating genome-level genetic selection.

Based on these observations, we employed a combined method utilizing the *Fst* and *π*-*ratio* to screen for genetic selection signals in the SCS population, an approach widely used for analyzing environmental adaptation traits and genetic selection in various marine species [22,39,40]. KEGG enrichment analysis identified several broad biological pathways associated with environmental adaptation, including taste transduction, melanogenesis and gastric acid secretion in the SCS vs. RS population and autophagy in the SCS vs. AS population. Through our screening process, we identified 21 overlapping genes in the selection regions of SCS vs. AS and SCS vs. RS comparisons. These genes represent potential candidates involved in climate adaptation within the SCS population. Functional annotation ultimately revealed five key genes related to environmental adaptation: *myo5a*, *hps4*, *znf385a*, *msh3*, and *pfkfb4*.

Melanin plays a crucial role in UV light absorption, reducing damage to skin and tissues, and its adaptive function strongly correlates with climate change impacts [41,42,43]. The *myo5a* gene interacts with melanosomes through the RAB27A/MLPH receptor in melanocytes [44], and its direct link to heat stress has been confirmed through genome-wide association analyses [45]. Additionally, *hps4* activates Rab32/38, key functional factors in melanocytes that participate in crucial steps of melanin synthesis [46]. Climate change-induced temperature fluctuations affect water oxygen levels, implicating hypoxia-associated genes in coastal species’ environmental adaptation. The *pfkfb4* gene, a target of hypoxia-inducible factor 1-alpha (HIF1A), is essential for hypoxia-induced glycolysis [47,48] and can modulate autophagy through ROS level regulation [49]. DNA damage repair capabilities also play a crucial role in environmental adaptation. The *znf385a* gene, situated upstream of the p53 activation pathway, promotes cell cycle arrest induced by DNA damage [50], while certain polymorphisms in *msh3* may influence DNA repair capabilities [51].

Our study provides valuable genomic resources regarding *H. kelloggi*’s environmental adaptation-related selection features across different regions, offering important insights for future research into these genes’ functional roles in adaptive evolution. However, the limited number of populations and sample sizes may introduce some bias in the genetic diversity estimates. Future studies should analyze *H. kelloggi* genetic diversity across broader geographic areas with larger sample sizes.

## 4. Materials and Methods

### 4.1. Ethics Statement

All sampling and procedures involving *H. kelloggi* were performed in accordance with the Wild Animals Protection Law of the People’s Republic of China and approved by the Animal Ethics Committee of the Chinese Academy of Sciences (approval number: SCSIO-IACUC-2019-000137). All individuals were treated with MS-222 (50 mg/L) before experimental treatment.

### 4.2. Samples Collection

**Figure 6:**
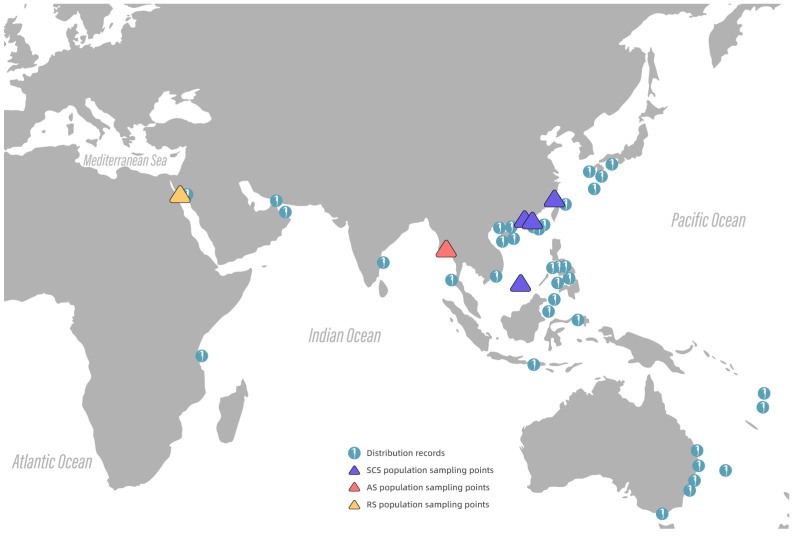
Information on the natural distribution of *H. kelloggi* and sampling sites. The *H. kelloggi* pattern map indicates their natural distribution density, and the blue, red, and yellow triangles represent the population sampling points for the SCS, AS, RS.

Fifty-six individuals of *H. kelloggi* from different seas were collected for use in this study, including 30 individuals from the Red Sea (RS), 13 individuals from the Andaman Sea (AS), and 13 individuals from the South China Sea (SCS). The distribution of sampling points is referenced from the following link: https://www.gbif.org/species/5201145 (accessed on 30 January 2025). From each population, 2–3 individuals were selected in order to amplify and sequence their COI genes. The sequences obtained were used for multiple sequence alignments and visualization using the Weseq tool from the WeMol computing platform (https://wemol.wecomput.com), and all of them were identified as *H. kelloggi* (Figure S2). The template sequences for comparison were obtained from the Barcode of Life Data System database (https://www.boldsystems.org). Information about the geographic location of all samples in this study and the distribution density of the *H. kelloggi* is shown in Figure 6.

### 4.3. DNA Extraction and Whole-Genome Re-Sequencing

The fin tissue from each collected sample was used to extract high-quality genomic DNA using the CTAB method. DNA purification was performed using the QIAGEN^®^ kit (Cat#13343; QIAGEN, Hilden, Germany), and DNA integrity was checked through agarose gel electrophoresis. The DNA concentration was accurately quantified using the Qubit v4.0 (Invitrogen, Waltham, MA, USA). Subsequently, the whole-genome DNA was sent to Wuhan Zhenyue Biotechnology Co., Ltd. (Wuhan, China) for DNA library construction based on the Illumina NovaSeq platform. The raw data were processed using FASTP v0.12.4 software (http://www.bioinformatics.babraham.ac.uk/projects/fastqc (accessed on 30 January 2025)) to remove adapter contamination, low-quality sequences, and reads with a high N content (≥10%). After initial quality control, clean reads were aligned to the seahorse reference genome using BWA v0.7.17-r1188 software (https://github.com/lh3/bwa/releases/tag/v0.7.17 (accessed on 30 January 2025)) [52]. Duplicates reads were then removed using Picard software (https://www.psc.edu/index.php/user-resources/software/picard (accessed on 30 January 2025)). The deduplicated data were used for statistics on alignment rate, coverage, and sequencing depth. GATK v4.2.2.0 (https://gatk.broadinstitute.org/hc/en-us/sections/4405443482011-4-2-2-0 (accessed on 30 January 2025)) was employed for variant detection and filtering, resulting in high-confidence SNPs [53]. VCFtools v0.1.16 software (https://vcftools.github.io/man_latest.html (accessed on 30 January 2025)) was utilized for SNP filtering, and the detected SNP loci in the samples were annotated using ANNOVAR software (https://annovar.openbioinformatics.org/en/latest/user-guide/download/ (accessed on 30 January 2025)) [54]. Finally, to ensure the reliability of subsequent population analyses, SNPs were filtered at the population level based on the following criteria: (1) minimum allele frequency (MAF) > 0.05; (2) the ratio of samples containing SNPs to the total number of samples (SNP call rate) > 80%.

### 4.4. Genetic Diversity Statistics

Genetic diversity indices for the three seahorse populations were calculated using PLINK v1.90 (https://www.cog-genomics.org/plink/ (accessed on 30 January 2025)) with default parameters. The analyzed parameters included the *Pic*, where *Pi* and *Pj* represent the frequencies of the *i*-th and *j*-th alleles, respectively; *He*; *Ho*, calculated as the ratio of heterozygous genotypes to total individuals; *Ao*, defined as the count of distinct alleles at each locus; *Ae*; and *Fis*. Population differentiation was evaluated using VCFtools with default parameters to calculate the *π*-*ratio* and *Fst*. Following Wright’s criteria [55], population differentiation was classified as low (*Fst* < 0.05), moderate (0.05 < *Fst* < 0.15), high (0.15 < *Fst* < 0.25), or extremely high (*Fst* > 0.25). *Nm* was estimated using the formula *Nm* = [(1/*Fst*) − 1]/4.

### 4.5. Population Structure Analysis

Based on the completely filtered SNP data, we used a principal component analysis and the method of constructing a phylogenetic tree to explore the group stratification to confirm the genetic evolutionary relationship between individuals and groups. A principal component analysis (PCA) was conducted using PLINK v1.90. The phylogenetic tree of the sample population was constructed using the maximum likelihood (ML) algorithm of RAxML software (https://github.com/stamatak/standard-RAxML (accessed on 30 January 2025)) [56,57]. Subsequently, the Admixture v1.3.0 software (https://biodockerfiles.github.io/admixture-1-3-0/ (accessed on 30 January 2025)) was used to assess the population structure of all samples [58]. The tested *K* values (subgroup numbers) were set from 1 to 5, and the optimal *K* value was determined by the lowest cross-validation error.

### 4.6. Historical Effective Population Size

SMC++ is a statistical inference method based on coalescent theory. It leverages genomic SNP data from multiple individuals and employs a Markov Chain Monte Carlo (MCMC) approach to analyze the coalescent patterns of gene lineages, thereby inferring a time series of *Ne* and characterizing the historical effective population size [59]. We employed the SMC++ v1.15.2 (https://github.com/popgenmethods/smcpp/releases (accessed on 30 January 2025)) method to estimate the *Ne* of three populations [58]. Use the vcf2smc script embedded in SMC++ to convert each VCF file to the required input file format. All simulations were performed with the default mutation rate set at 1.25 × $10^{-8}$ as the initial condition.

### 4.7. Linkage Disequilibrium (LD) Decay Assay

An *LD* analysis was performed using PopLDdecay v3.41 (https://github.com/BGI-shenzhen/PopLDdecay/releases (accessed on 30 January 2025)) to calculate pair-wise SNP associations and generate *LD* decay plots. The R-squared correlation coefficient ($r^{2}$) was calculated using the formula $r^{2}$ = D^2^/(PA·Pa·PB·Pb), where D represents the deviation of allelic frequencies at two loci from random association and PA, Pa, PB, and Pb denote the frequencies of alleles A, a, B, and b, respectively. The *LD* decay plots display the $r^{2}$ values against the physical distance between pair-wise SNPs in the reference genome. We defined the *LD* decay distance as the genomic physical distance at which the *LD* value decreases to half of its maximum.

### 4.8. Screening for Selective Sweeps

In order to compare the genetic differences between seahorse populations from different geographical regions, we conducted joint *π*-*ratio* and *Fst* tests to detect potential selection signatures on the genome using a window size of 5 kb and a step size of 5 kb. The AS and RS populations were designated as control groups, while the SCS population was designated as the experimental group. Based on the results of the genetic parameters, the top 5% of windows for the *Fst* values and *π*-*ratio* were selected as signal regions for strong selection to be used for subsequent candidate gene localization and functional enrichment analyses.

### 4.9. Identification of the Candidate Genes Associated with Selection Signatures

The identified genomic selection signals were mapped to the annotation file of the seahorse genome to determine gene function [60]. Subsequently, the genes were subjected to GO and KEGG enrichment analyses to identify the biological processes and pathways in which candidate genes may be involved.

## 5. Conclusions

This study conducted WGRS on three populations of *H. kelloggi* from the RS, the AS, and the SCS, identifying 1,398,936 high-quality SNPs. A genetic diversity analysis shows that, among the three populations, the SCS population has higher genetic diversity, while the AS population has lower genetic diversity. A genetic differentiation analysis revealed significant differentiation among these geographic populations, with limited gene flow. A population structure analysis clearly distinguished these groups, consistent with the results of the population differentiation coefficient. A demographic analysis indicated that the SCS population experienced a significant bottleneck during the LGM, followed by recovery, while the *Ne* of the RS and AS populations remained stable. Through a selective sweep analysis, 21 candidate genes potentially involved in the environmental adaptation of the SCS population were identified, notably *myo5a*, *hps4*, *znf385a*, *msh3*, and *pfkfb4*, which may play key roles in the adaptive mechanisms of *H. kelloggi*. This comprehensive study not only elucidates the genetic diversity patterns of *H. kelloggi* but also provides a crucial foundation for future research on the species’ evolutionary adaptation.

## Figures and Tables

**Table 1:** SNP annotation by genomic region and function class.

Category	Type (Alphabetical Order)	Count	Percentage (%)
Region	upstream (1 kb)	2,293,268	3.514
	exonic	2,608,787	3.918
	intronic	31,282,950	46.987
	intergenic	26,657,611	40.04
	5′UTR	328,690	0.494
	3′UTR	1,209,413	1.817
	downstream (1 kb)	2,190,317	3.290
	upstream; downstream	357,404	0.527
	splicing	11,268	0.017
Function class	synonymous SNV	1,433,592	2.153
	nonsynonymous SNV	1,155,340	1.735
	stopgain	10,658	0.016
	stoploss	2293	0.003

**Table 2:** Summary of genetic diversity compared between the Red Sea (RS), the Andaman Sea (AS), and the South China Sea (SCS) populations.

Pop	*Ho*	*He*	*Pic*	*Fis*	*Ao*	*Ae*	*π-Ratio*
RS	0.2031	0.1987	0.1673	−0.0082	1.6178	1.3694	0.3269
AS	0.1914	0.1822	0.1534	−0.0251	1.5646	1.3358	0.3353
SCS	0.2083	0.2001	0.1654	−0.0184	1.5660	1.3824	0.3677

*Ho*, average observed heterozygosity; *He*, average expected heterozygosity; *Pic*, polymorphism information content; *Fis*, inbreeding coefficient; *Ao*, observed number of alleles; *Ae*, effective number of alleles; *π*-*ratio*, nucleotide diversity ratios.

**Table 3:** Genetic differentiation analysis among three *H. kelloggi* populations.

Group	RS	AS	SCS
RS	0	0.2371	0.3222
AS	0.8045	0	0.3183
SCS	0.5258	0.5355	0

Pairwise fixation index (*Fst*) value (top right) and the number of effective migrants (*Nm*) value (bottom left).

**Table 4:** Candidate genes related to regional environmental adaptation in the SCS population.

LG	Start (bp)	End (bp)	Gene ID	Gene Symbol	*Fst* (CHN and THA)	*Fst* (CHN and EGY)	*π-Ratio* (THA/CHN)	*π-Ratio* (EGY/CHN)
4	5105001	5110000	Hke018906		0.5662	0.4900	3.854	5.265
4	5325001	5330000	Hke018922	*uckl1*	0.7035	0.5425	4.885	20.383
6	4915001	4920000	Hke019774	*cyc1*	0.6107	0.4928	7.782	9.077
10	15875001	15880000	Hke014228	*prkd3*	0.6371	0.6408	15.632	12.435
12	10630001	10635000	Hke006341	*adam22*	0.5889	0.4953	10.243	12.111
15	740001	745000	Hke004708	*ythdc2*	0.7045	0.6342	4.800	4.958
15	13345001	13350000	Hke005245	*msh3*	0.5599	0.6784	10.262	9.447
15	19335001	19340000	Hke005537	*hps4*	0.5676	0.5120	7.629	10.824
16	1055001	1060000	Hke006809	*pfkfb4*	0.6299	0.5318	4.407	6.020
16	16090001	16095000	Hke007537		0.6363	0.5591	27.982	34.741
16	16655001	16660000	Hke007578	*rbm12*	0.7005	0.5678	26.760	24.788
17	11870001	11875000	Hke004222	*myo5a*	0.5841	0.5080	6.895	5.935
17	22660001	22665000	Hke004577	*hydin*	0.5644	0.6902	10.742	6.254
18	24365001	24370000	Hke010048	uncharacterized gene	0.5893	0.5880	15.675	13.745
	24370001	24375000			0.5829	0.6038	22.150	15.978
	24385001	24390000			0.5745	0.5744	8.127	5.582
	24400001	24405000			0.5801	0.5901	9.379	8.319
	24405001	24410000			0.6603	0.5688	16.987	13.649
18	24405001	24410000	Hke010049	uncharacterized gene	0.6603	0.5688	16.987	13.649
18	24540001	24545000	Hke010050	uncharacterized gene	0.5994	0.4970	6.500	7.031
18	24610001	24615000	Hke010051	*mhc*	0.6803	0.6164	23.842	25.619
	24615001	24620000			0.6086	0.5151	16.228	15.089
19	2930001	2935000	Hke012808	*actr3*	0.5733	0.5279	11.117	11.360
20	9625001	9630000	Hke001750	*efnb3*	0.5354	0.5002	12.897	12.440
21	19560001	19565000	Hke000631	*szrd1*	0.6442	0.5060	3.969	5.247
21	33685001	33690000	Hke001234	*znf385a*	0.5629	0.5928	5.264	5.851

LG, linkage group; Start, start physical location of genes on the reference genome; End, end physical location of genes on the reference genome; *Fst*, pairwise fixation index; *π*-*ratio*, nucleotide polymorphisms.

## Data Availability

The raw reads for the *H. kelloggi* individuals have been deposited in the NCBI Sequence Read Archive under the accession number PRJNA1136163.

## Supplementary Materials

The following supporting information can be downloaded at: https://www.mdpi.com/article/10.3390/ijms26031387/s1.
