# Supplementary material for: Genetic Diversity and Environmental Adaptation Signatures of the Great Seahorse (Hippocampus kelloggi) in the Coastal Regions of the Indo-Pacific as Revealed by Whole-Genome Re-Sequencing
Source: Int J Mol Sci. 2025 Feb 6;26(3):1387. doi: 10.3390/ijms26031387 (PMC11818898; doi:10.3390/ijms26031387)
Supplement: Supplementary file 1 [file ijms-26-01387-s001.zip › Supplementary Figure S1 S2 .docx]

**Figure S1. Phylogenetic tree of four groups within SCS population.**

**
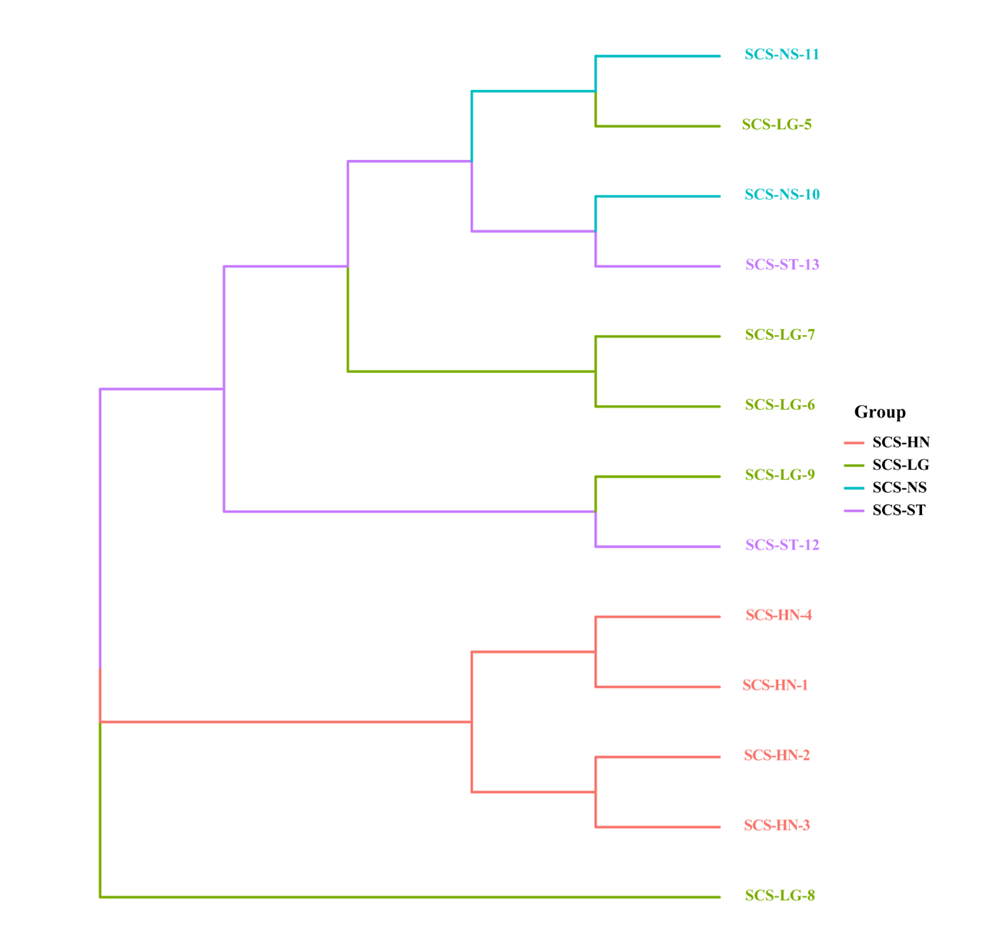
**

**Figure S2. Visualization of partial sequence alignment results**

**
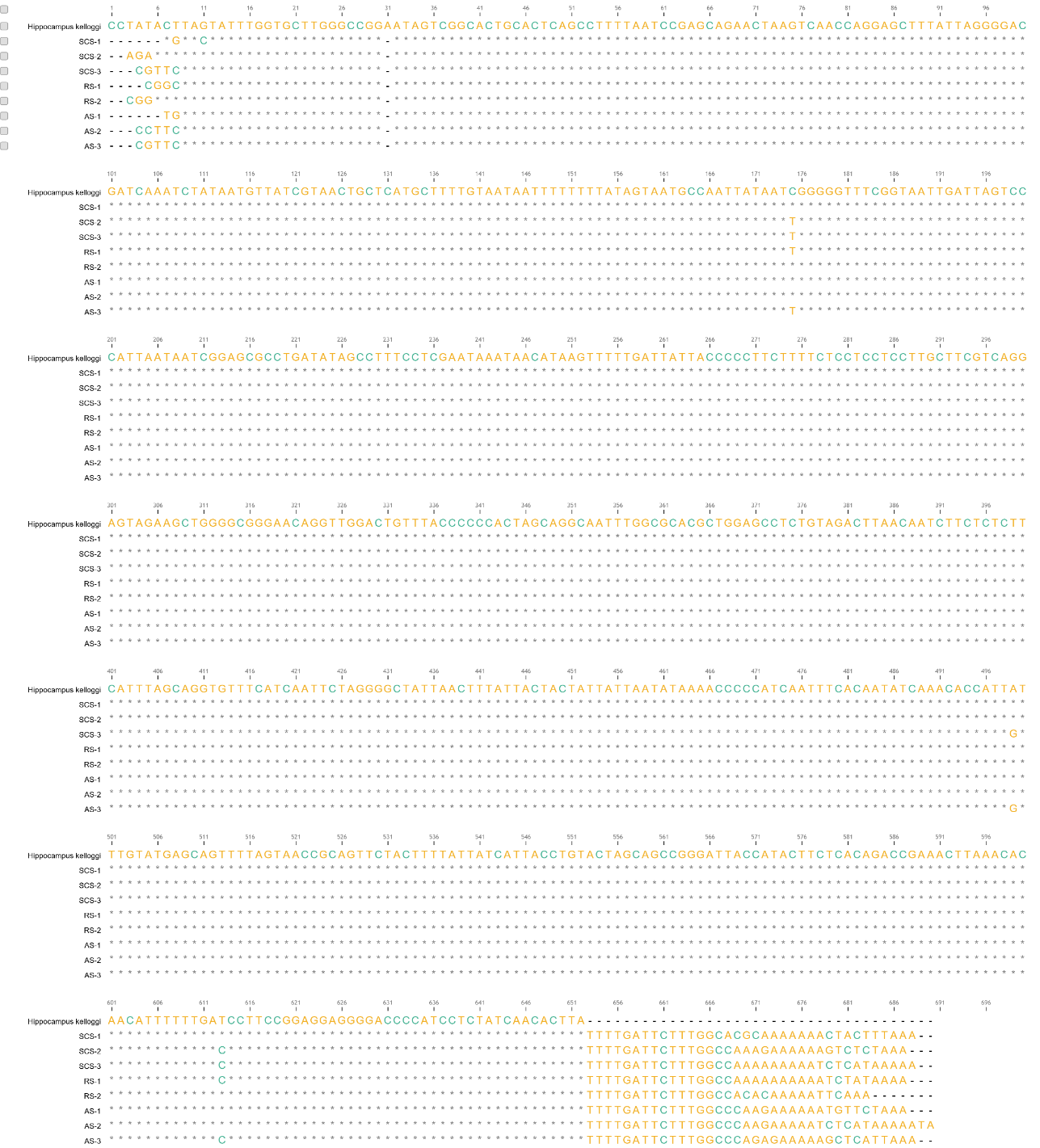
**
